# Diagnosing awareness in disorders of consciousness with gamma-band auditory responses

**DOI:** 10.3389/fnhum.2023.1243051

**Published:** 2024-01-05

**Authors:** Marek Binder, Julia Papiernik, Inga Griskova-Bulanova, Sandra Frycz, Bartłomiej Chojnacki, Urszula Górska-Klimowska

**Affiliations:** ^1^Institute of Psychology, Jagiellonian University, Kraków, Poland; ^2^Doctoral School in the Social Sciences, Jagiellonian University, Kraków, Poland; ^3^Life Sciences Centre, Institute of Biosciences, Vilnius University, Vilnius, Lithuania; ^4^Department of Mechanics and Vibroacoustics, Faculty of Mechanical Engineering and Robotics, AGH University of Krakow, Kraków, Poland; ^5^Department of Psychiatry, University of Wisconsin-Madison, Madison, WI, United States

**Keywords:** disorders of consciousness, consciousness, EEG, auditory stimulation, auditory steady-state responses, envelope following response, Coma Recovery Scale-Revised

## Abstract

**Introduction:**

The prolonged disorders of consciousness (pDOC) describe a group of neurological conditions characterized by severe impairment of consciousness resulting from the injury of the central nervous system. As the behavioral diagnosis of pDOC remains challenging, the methods based on observing brain activity appear as promising alternatives. One of these methods is electroencephalography, which allows for noninvasive assessment of brain function.

**Methods:**

In this study, we evaluated evoked auditory responses to the chirp-modulated auditory stimulation as a potential biomarker of awareness in pDOC. Chirp-modulated stimulation is based on the repetitive presentation of auditory stimuli with a changing frequency over time. Two protocols were tested: amplitude-modulated narrow-band chirps (frequency range 25–55 Hz) and click-based wide-band chirps (30–100 Hz). The studied pDOC patient group included 62 patients (19 females and 43 males, mean age 40.72 years) diagnosed with Coma Recovery Scale-Revised. Envelope-following responses to stimulation were examined using the intertrial phase clustering coefficient.

**Results:**

For both types of stimulation, the strength of the response in the low-gamma range (around 40 Hz) was related to the diagnosis of pDOC. Patients diagnosed with unresponsive wakefulness syndrome exhibited diminished responses, while more favorable diagnoses, suggesting awareness (minimally conscious state or emergence from the minimally conscious state), showed elevated responses. The variations in the integrity of the auditory pathway and the etiology of brain injury altered the observed response strength. Narrow-band stimulation yielded a more systematic relationship between low-gamma response and pDOC diagnosis.

**Discussion:**

The results suggest the potential role of low gamma-band responses to chirp-modulated stimulation as the supportive diagnostic tool to detect awareness in the pDOC patient group.

## 1 Introduction

The prolonged disorders of consciousness (pDOC) include the group of neurological conditions that result from extensive damage to the neuronal tissue of the central nervous system. The causes of such disorders vary, with traumatic brain injury (TBI) and anoxia being the most frequent ones (Estraneo and Trojano, 2018). pDOC include conditions, such as unresponsive wakefulness syndrome (UWS, also known as vegetative state; Laureys et al., 2010) and minimally conscious state (MCS; Giacino et al., 2002), which is divided into subdiagnoses of minimally conscious state minus (MCS-) and minimally conscious state plus (MCS+; Thibaut et al., 2020). MCS+ is recognized when a patient displays signs of communication skills (e.g., command following), and MCS- when only non-verbal symptoms of consciousness can be observed (e.g., visual pursuit). The pDOC patients who have regained consciousness are diagnosed with emergence from a minimally conscious state (EMCS; Giacino et al., 2002).

Even though various diagnostical tools exist, they lack sufficient diagnostic accuracy, as approximately 40% of patients with UWS are misdiagnosed (Schnakers et al., 2009). In recent years, various methods based on observation of brain activity were proposed to tackle that issue, including, among others, functional magnetic resonance imaging (fMRI), positron emission tomography (PET), and electroencephalography (EEG), used in isolation or with concurrent transcranial magnetic stimulation (TMS-EEG technique; Giacino et al., 2018; Kondziella et al., 2020). Among those techniques, the potential diagnostical role of auditory steady-state response (ASSR) has been evaluated (Binder et al., 2017; Górska and Binder, 2019). This method seems a suitable alternative for DOC patients, being relatively cheap, robust, and technically less challenging than fMRI PET or TMS-EEG. It might be especially beneficial when clinical scales such as Coma Recovery Scale–Revised give ambiguous results due to an extensive motor or visual dysfunction. The ASSRs were analyzed as trains of clicks or amplitude-modulated sounds with constant stimulation frequency and as trains of periodic stimuli with chirp-modulated variable frequency (Binder et al., 2020). The latter solution allows for inducing oscillations in broader spectra of frequencies. In stimulations spanning the range of at least 30–100 Hz, two peaks of heightened responsitivity are often detected (Artieda et al., 2004; Pipinis et al., 2018). The first is centered around 40 Hz (labeled as the low-gamma band response), and the second is around 80–100 Hz (labeled as the high-gamma band response). The evoked activity in both of these frequency ranges has a different distribution of primary sources, with low-gamma response mainly originating in cortical, thalamic, and brainstem sources, while the high-gamma response predominantly generated by the brainstem sources with a lesser contribution from the higher levels of the auditory pathway (Herdman et al., 2002; Farahani et al., 2017, 2019, 2021). Applying ASSR-based protocols to pDOC patients revealed the promising correlation between the level of consciousness and the phase-locking index (PLI) in the low-gamma range (Binder et al., 2017, 2020; Górska and Binder, 2019). However, those studies were based on relatively small groups of patients, thus requiring further research to confirm the initial results.

The current study aimed to explore responses to chirp-modulated sounds in the low and high gamma ranges as potential biomarkers of awareness in pDOC on a larger patient sample. The response to two types of chirp stimulation was evaluated, and the intertrial phase clustering coefficient (ITPC) was analyzed for the chosen ranges of the responses. The Polish version of the Coma Recovery Scale–Revised (CRS-R; Giacino et al., 2004; Binder et al., 2018) was used as a reference for the pDOC diagnosis. In the previous study (Binder et al., 2020), we found that the low-gamma response to periodic auditory stimulation displays sensitivity to the condition of the pDOC patients as measured with CRS-R.

## 2 Materials and methods

### 2.1 Subjects

The convenience sample of pDOC patients consisted of 62 subjects, 19 females and 43 males (31% females and 69% males), with a mean age of 40.72 (SD = 12.91, range from 18 to 74 years old); one subject was left-handed, and one was ambidextrous. The sample of healthy control (HC) consisted of 20 subjects, 9 females and 11 males (45% females and 55% males), mean age of 29.45 (SD = 9.1, range from 20 to 55 years old), two subjects were left-handed. The mean ages in groups were compared using two-tailed *t*-test for unequal variances and received *p*-value < 0.0001, indicating that mean age differed significantly between the patient and control groups. The gender ratios were compared between the control and patient groups using the Fisher’s Exact Test, which resulted in an insignificant result of *p* = 0.283, indicating that gender ratios did not differ significantly between the groups.

The control group was studied between February 2020 and September 2021, and the patient group between December 2020 and February 2023. For each subject, an informed consent was acquired. In the case of the participants from the pDOC group, the consent was given by their legal surrogates. The study design was approved by the local review board at the Institute of Psychology, Jagiellonian University, Kraków, Poland, and followed the provisions of the Declaration of Helsinki. The patients who took part in our study received a standard clinical treatment for patients diagnosed with prolonged disorders of consciousness, which involved physical therapy, pharmacotherapy, speech therapy, and general patient care treatment. The specific regimen of those clinical interventions depended on each patient’s individual needs.

For the control group, the exclusion criteria were the presence of mental or neurological problems and pharmacological treatment with psychoactive medications. Inclusion criteria involved passing the audiological screening test set.

For the pDOC patient group, the inclusion criteria included: diagnosis of the prolonged disorder of consciousness (unresponsive wakefulness syndrome, minimally conscious state ±, emergence from the minimally conscious state), age 16–80 years, acquired severe brain injury, and passing the audiological screening test set (the details of screening procedure are described below). The exclusion criteria included: severe somatic conditions influencing pDOC diagnosis and EEG activity (e.g., severe hepatic or renal insufficiency, seizure activity during EEG acquisition) and schizophrenia before the incident causing pDOC. Patient studies were conducted in the rehabilitation centers located in Poland: PCRF “Votum” centers in Kraków and Sawice, COiR “Zdrowie” Center in Czȩstochowa, and Fundacja “Światło” Center in Toruń.

Both groups underwent an audiological screening test set with the use of Titan device v. 3.4.1 (Interacoustics A/S, Middelfart, DK), testing integrity of the inner ear with otoacoustic emissions and integrity of the auditory pathway with auditory brainstem responses. The chosen screening protocols for otoacoustic emissions included Transient Evoked Otoacoustic Emissions (TEOAE) testing, based on a repeated broad-band click stimulus, activating a wide area of the basilar membrane, and Distortion Product Otoacoustic Emissions (DPOAE), which use the simultaneous presentation of two pure- tones to evoke and measure the distortion that occurs in various places along the cochlea. The hearing of 1000, 1500, 2000, 3000, and 4000 Hz frequencies was investigated using two TEOAE protocols. Two DPOAE protocols were used: the first examined the hearing of 500, 594, 707, 840, and 1000 Hz frequencies, and the latter focused on 500, 1000, 1500, 2000, 3000, 4000, 5000, 6000, 7000, 8000, 9000, and 10000 Hz frequencies. A threshold was set for at least three frequencies per protocol to meet the “pass” criterion for a protocol to be considered passed. The only exception was made for the second DPOAE protocol, in which 7 out of 12 measured frequencies were required to meet the “pass” criterion for the protocol to be considered passed. The screening test for the integrity of the auditory pathway was based on ABR (auditory brainstem response) measurement. It involved the proprietary CE-Chirp^®^ ABRIS screening test protocol with 35 dBnHL sound intensity with the standard mastoid montage, two electrodes placed on mastoids, and one on the forehead of the participant. The response was displayed as “pass” or “refer.” The final inclusion screening criterion required for participants to be included in the study was passing at least one of the otoacoustic emission tests and/or passing the ABR screening test. Only patients who passed this criterion were included in EEG data analysis. Datasets of some of the patients were further discarded during preprocessing due to low signal quality or technical problems encountered during signal acquisition. The final sizes of patient groups included in the data analysis are provided in Table 1. The tables containing detailed information about controls and patients can be found in the Supplementary Table 1 (controls) and Supplementary Table 2 (patients).

**TABLE 1:** The number of observations/subjects included in final analyses in both experimental conditions, split by the most frequent diagnosis.

Condition	UWS	MCS-	MCS+	EMCS	Total
NBC	28	15	6	5	54
WBC	27	11	3	5	46

NBC, narrow-band chirp condition; WBC, wide-band chirp condition; UWS, unresponsive wakefulness syndrome; MCS, minimally conscious state minus; MCS+, minimally conscious state plus; EMCS, emergence from the minimally conscious state.

All patients were assessed using the Polish version of the CRS-R scale (Binder et al., 2018) for the pDOC diagnosis. Each patient was evaluated by at least two different examiners. Five CRS-R assessments per patient were done within a week. The total score, subscale scores, and the diagnosis were noted for each assessment. During the evaluation, patients were either seated in a wheelchair or raised in their beds to be in an upright position. The background noises, such as TV or radio, were muted for the time of the administration.

### 2.2 Stimuli

**FIGURE 1:**
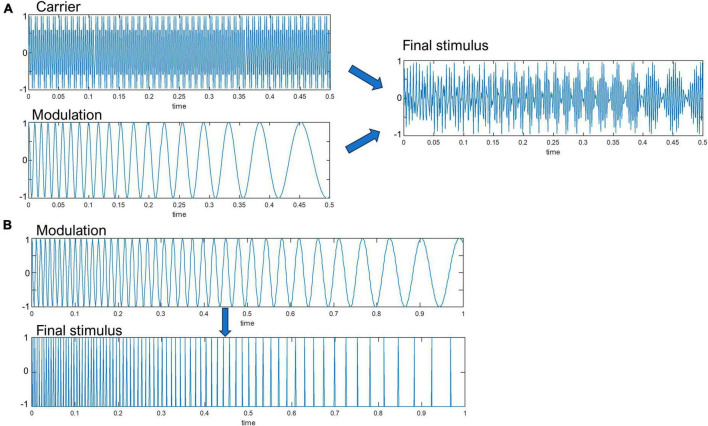
A schematic representation of the **(A)** narrow-band amplitude-modulated, **(B)** wide-band click-based chirp stimulus. Note that the white-noise bursts were emitted at the zero-crossings of the modulation waveform. The *Y*-axis represent sound pressure level in arbitrary units.

The auditory stimuli were designed in the MATLAB environment (The MathWorks, Inc.). Two types of auditory stimuli were created: narrow-band chirp-modulated and wide-band chirp-modulated sounds. Each individual narrow-band chirp-modulated stimulus consisted of 1000 Hz carrier tone 100% amplitude modulated with a linear chirp that decreased in frequency from 55 to 25 Hz during 500 ms time (see Figure 1A). Stimulus duration was 500 ms, with 10 ms onset/offset linear ramps to avoid onset and offset clicks. Wide-band chirp-modulated stimuli were a series of single clicks 1 ms white-noise bursts distributed in a logarithmic manner, which decreased in frequency from 100 to 30 Hz during 1000 ms time (see Figure 1B). Both types of stimuli were presented at the sound intensity of 60 dB.

An acoustic calibration procedure was conducted to ensure an accurate and stable sound pressure level (SPL) is present. The performed test was prepared to verify two factors: firstly, the stability of the acoustic output measured inside the ear, and secondly, the repeatability of the measured SPL regarding the in-ear pads used with the consideration of difference for trials on put on and put off inside the ear.

The acoustic measurement test was conducted in an anechoic chamber of AGH University of Kraków with the Bruel and Kjaer type 4128-C Head and Torso simulator (HATS) with artificial ear and built-in microphones connected to two SVAN 912E sound meters. Each stimulus was measured five times after the calibration to verify if it was possible to achieve stable SPL. The LAeq sound level was measured within the 10-s time frame. Previous work defined proper binaural stimuli testing level as 65 dB SPL (Neher et al., 2017) or 60 dB SPL with EEG testing (Ignatious et al., 2021). In this work, the base level was set as 60 dB with the active weighting curve A (dBA) as it better reflects the actual human hearing mechanism and was proved to be the proper level of long-term brain stimuli testing (Kasprzak, 2011). The results of the acoustic testing procedure are presented in the Supplementary Table 3.

All stimuli levels were properly calibrated around 60 dBA. The standard deviation from the five measurement trials in all cases was lower than 1 dB, which was the result claiming good repeatability between measured subjects (Engel, 2001).

### 2.3 Experimental procedure

Each participant was presented with 300 repetitions of wide-band chirp-modulated sounds with 2220–3020 ms variable inter-stimulus intervals (in 200 ms steps) in the wide-band chirp condition (hence labeled WBC) and 300 narrow-band chirp-modulated sounds stimulus repetitions with 1220–1520 ms variable inter-stimulus intervals (in 100 ms steps) in the narrow-band chirp condition (hence labeled NBC), in a fixed order. Control subjects were evaluated in the sleep laboratory while seated on the bed, with eyes open, alone in a separate room with dimmed lights. During patient studies, all participants were placed in a wheelchair or remained in their beds in an upright position. Recording occurred in a separate room or the patient room, with only the patient and two experimenters present. Ambient noise levels were not monitored at either recording session. EEG acquisition was performed when patients had their eyes open to ensure they were awake. Experimenters were blind to the results of the final CRS-R diagnoses at the time of recording and data processing.

### 2.4 Apparatus

Auditory stimuli were delivered using ER-3C insert earphones (Etymotic Research, Elk Grove Village, IL, USA) and a headphone amplifier Millenium HP1. EEG recordings were conducted using a 64-channel Active Two system (BioSemi, Amsterdam, Netherlands), with a 10–20 system head cap and four additional leads located above and below the right eye and in the external canthi of both eyes. Two added reference electrodes were placed on mastoids and recorded in parallel. CMS and DRL electrodes were placed between POz and PO3 and POz and PO4, respectively. Data were sampled at 1024 Hz. Stimulus presentation was controlled by Presentation software (Neurobehavioral Systems, Berkeley, CA, USA). The audio signal was recorded concurrently with EEG data using Analog Input Box (Biosemi, Amsterdam, NL, USA) and stored in a single dataset. The synchronization between the onset of the auditory stimulation and temporal markers in the EEG data indicating the start of the stimulation was verified off-line before data preprocessing steps.

### 2.5 Data processing

The initial preprocessing steps were performed using Brain Vision Analyzer 2.2 (Brain Products, Gilching, DE, USA). During the first step, data were filtered using an IIR high-pass filter (Zero phase shift Butterworth filter, eight order) and notch filter (at 50 Hz). Then, data were re-referenced to a common average reference and downsampled to 512 Hz. Noisy channels (e.g., muscle artifacts, loss of contact) were rejected and further interpolated. Eye movement correction was performed using the ICA ocular correction module (Independent Component Analysis) implemented in Brain Vision Analyzer 2 software and a semi-automatic module for blink detection. For further analysis, seven frontocentral channels were selected (FC1, FC2, C1, C2, Fz, FCz, Cz), as these regions display the most robust response to periodic auditory stimulation (Schwarz and Taylor, 2005; Spencer et al., 2008; Voicikas et al., 2016) and are less susceptible to artifacts. The continuous EEG data from the selected datasets were segmented into −700, 1200 ms epochs in the narrow-band chirp condition and into −700, 1700 ms epochs in the case of the wide-band chirp condition. In the next step, all individual epochs in both conditions were baseline-corrected using a pre-onset period −699, −200 ms. After that, segments containing artifacts were rejected using semi-automatic mode with the following criteria: amplitude limits −200 μV to 200 μV; 200 μV maximum allowed difference in intervals over 200 ms; maximal voltage step of 150 μV/ms. Using custom MATLAB (The MathWorks, Inc.) scripts employing FieldTrip functions (Oostenveld et al., 2011), the number of epochs across subjects was equalized using the following rule: the minimum number of epochs necessary for further analysis was set to 200 epochs, and if the number of epochs exceeded 240, this number of epochs was randomly selected from the available set. This step resulted in the rejection of some datasets, and the final group sizes are shown in Table 1.

In the next step, time-domain data were decomposed into time-frequency representation using FieldTrip function ft_freqanalysis, with short-term Fourier transformation and Hanning taper (using mtmconvol option with Hanning taper), with the following transformation settings: time-window 500 ms, bandwidth 2–120 Hz, with 2 Hz steps, output temporal resolution 9.765625 ms. Using a custom MATLAB script, the TF data were then used to calculate the ITPC (also known as a phase-locking index, PLI). The ITPC was calculated using the following formula (based on Delorme and Makeig, 2004 and FieldTrip documentation):

$$I\,T\,P\,C\,\left(f,t\right)=\left|\frac{1}{n}\,\sum_{k=1}^{n}\frac{F_{k}\,\left(f,t\right)}{\left|F_{k}\,\left(f,t\right)\right|}\right|$$

Where F_k_(f,t) is the spectral estimate of trial k at frequency f and time t, and n is the number of trials.

### 2.6 Data analysis

**FIGURE 2:**
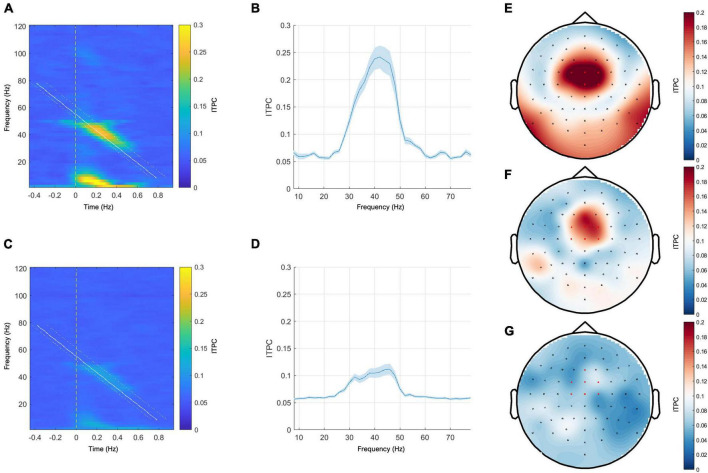
Left panel: grand mean responses to narrow-band chirp-modulated stimulation on the time-frequency plane in the healthy control group **(A)** and in the patient group **(C)**. Middle panel: grand mean EFR curves over frequency in the healthy control group **(B)** and the patient group **(D)**. The blue ribbons represent the standard error of the mean. Right panel: topoplots for the narrow-band chirp condition ITPC response sampled from the range of 32–50 Hz. **(E)** Grand mean response of the control group, **(F)** representative subject with the MCS+ diagnosis and the positive ABRIS result, **(G)**—representative subject with UWS diagnosis and the positive ABRIS result. Red-colored dots indicate channels that were included in calculating EFR responses.

The curve representing time-frequency points corresponding to the progression of chirp-modulated stimulation across time and frequency was used to estimate responses to chirp-modulated sounds. As we used periodic auditory stimuli that change their modulation frequency in time and consequently their envelope, we decided to use the term “envelope following response” (EFR; Dolphin, 1997) instead of “auditory steady-state response” to describe the observed evoked changes in the time-frequency domain of the EEG signal. Envelope following response is defined as the gross changes in the EEG signal caused by the populations of neurons that respond synchronously (phase-locked) to the envelope of an acoustic stimulus (Encina-Llamas et al., 2021), and in contrast to the ASSR definition, it does not assume the constant frequency of stimulation (Ross, 2013). The envelope following the frequency response curve (hence labeled EFR curve) was constructed using the MATLAB formula used previously to generate the stimulation. In order to estimate the prestimulus and post-stimulus level of the EEG signal, the envelope curve was extrapolated before onset and after the offset of the stimulus (see Figures 2, 5) and spanned the period −400, 800 ms for the narrow-band chirps and −160, 1190 ms for the wide-band chirps. To account for temporal smoothing due to the used method of time-frequency decomposition and the delay in sensory pathways, for each time-frequency point belonging to the envelope curve, the ITPC value was calculated at each frequency step as a mean of temporal window covering 50 ms before the stimulation and 100 ms after the onset of the stimulation (see the dashed lines in Figures 2, 5).

The individual CRS-R diagnoses of the patients were transformed into a single final diagnosis based on the most frequent diagnosis obtained by a patient during five assessments (the variable hence labeled FreqDiag). We did not choose to use the criterion of the best diagnosis to determine the patient’s condition during the study period because it is probable that such an approach may amplify the diagnostic error made during a single examination.

Due to the small and unequal sizes of MCS-, MCS+, and EMCS groups, exploring data for each diagnosis was impossible. To equalize the patient group size, MCS-, MCS+, and EMCS patients were combined to constitute the group of all pDOC patients who can be considered aware. This group was labeled “MCSe” (MCS “extended” group). Ultimately, two groups of participants were compared: UWS (presumably unaware subjects) and MCSe (presumably aware patients), with the HC group not included since it was used for identifying the shape and the localization of the EFR response.

To compare EFR response curves between these groups while effectively controlling the type I error in a situation involving multiple comparisons, we used a non-parametric cluster-based permutation procedure implemented in FieldTrip software (Oostenveld et al., 2011), using the same settings as the previously described analysis. We chose the ft_statfun_indepsamplesT function to estimate the statistical effects of that comparison. Samples that survived the initial test (i.e., the uncorrected *p*-value was less than 0.005) were clustered based on the temporal proximity. Cluster-level statistics were obtained by summing the sample statistics within each cluster. The maximum of these was used to evaluate the significance of the results against a randomization distribution. This distribution was obtained by randomly permuting the original data, taking the maximum cluster-level statistic (labeled as clusterstat in the section “3 Results”), and repeating this process 30,000 times. The probability of obtaining a statistic from this distribution larger than the actual cluster statistic was tested at a p-level set less than 0.001. We performed the one-sided test because our earlier studies provided evidence for higher ITPC responses in groups with more favorable CRS-R results (Binder et al., 2017, 2020).

To test for more specific effects based on the mean ITPC scores sampled from the frequency ranges of the suprathreshold clusters, we used the robust aligned rank transform ANOVA test where appropriate (with the p-level set at 0.05). These statistical analyses were conducted using jamovi software (Version 2.2.5; The Jamovi Project, 2022).

## 3 Results

### 3.1 Narrow-band chirp condition—Effects of diagnosis

The grand mean responses in the time-frequency domain and the grand mean EFR curves in the healthy control group and the patient group are shown in Figure 2. In both groups, the maximum ITPC response was observed between 40 and 50 Hz (see Figures 2B, D). The representative topoplots for the frequency range 32–50 Hz in Figure 2 (the right panel) show the maximum response at the frontocentral channels in the control group and in the representative case with MCS+ diagnosis, yet this response is barely visible in the representative patient from the UWS group.

Before further analysis, one outlier was removed from the NBC dataset due to an excessively high ITPC response. The outlier detection analysis was based on the interquartile range method applied to the whole envelope responses in the patient and control groups. We also included the control group in the outlier detection procedure since we expected that some responses, especially in the patients with EMCS diagnosis, might be comparable to the responses of healthy patients while being much higher than responses in the group of patients with UWS diagnosis.

**FIGURE 3:**
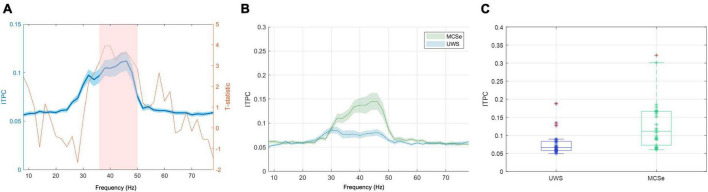
The narrow-band chirp condition results indicating differences in low-gamma response between aware and unaware groups (FreqDiag variable). **(A)** Results of the non-parametric cluster-level statistical analysis for the differences between MCSe and UWS groups, blue plot: grand average EFR response with overlaid T-statistic scores (red plot), the pink box represents the suprathreshold cluster, **(B)** mean EFR curves for both FreqDiag groups, **(C)** box-plots of group FreqDiag results with individual data, extreme values indicated in red. Ribbons on panels **(A,B)** represent the standard error of the mean.

The unaware group (UWS) consisted of 28 patients with the most frequent UWS diagnosis, and the aware group (MCSe) included 26 patients with the most frequent MCS-, MCS+, or EMCS diagnosis. We compared these two groups using a non-parametric cluster-based permutation procedure (see Figure 3A). We found a significant difference corresponding to a cluster at 36–50 Hz frequency range (clusterstat = 27.64, cluster significance *p* < 0.001), with higher response in the MCSe group (see Figure 3B). The distributions of individual ITPC scores (see Figure 3C) in both groups indicate that in the UWS group results are clustered from 0.05 to 0.01 scores with three cases above 0.1 level. The results of the MCSe group are on average higher, with several observations below 0.1 level. See Table 2 for the mean ITPC scores for both patient groups in this condition.

**TABLE 2:** Mean ITPC scores and standard deviations (in parentheses) for the suprathreshold clusters for the patient groups in all conditions.

Condition	UWS	MSCe
NBC	0.0749 (0.0296)	0.1199 (0.0606)
WBC	0.0749 (0.0324)	0.1322 (0.0549)

NBC, narrow-band chirp condition; WBC, wide-band chirp condition; UWS, the unaware group; MCSe, the aware group.

### 3.2 Narrow-band chirp condition—Effects of auditory pathway integrity

Our inclusion criteria allowed for patients with negative ABRIS screening test results. Such results indicate possible functional or structural disruptions of the brainstem part of the auditory pathway. There is evidence that such disruptions can decrease the strength of ASSR in the low-gamma band (Johnson and Brown, 2005) and thus introduce bias on the observed relation between pDOC diagnosis and EFR responses. First, to eliminate the factor of the integrity of the auditory pathway on the relation between EFR response and pDOC diagnosis, we repeated the non-parametric cluster-based analysis on the subset of the ABRIS-positive patients (i.e., those who have passed the ABR screening test). We found a significant difference in the same direction, corresponding to the single cluster at the 36–48 Hz frequency range (*N* = 40, clusterstat = 24.52, cluster significance *p* < 0.001), confirming that the observed relation is not dependent on the injuries of the brainstem part of the auditory pathway.

**FIGURE 4:**
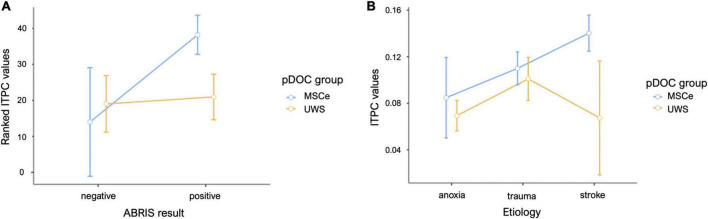
Mean plots for the data sampled from the suprathreshold cluster in the narrow-band chirp condition. **(A)** Mean results for the FreqDiag groups split by ABRIS screening test results. **(B)** Mean results for the FreqDiag groups split by etiology category. Error bars represent 95% confidence intervals.

To further explore the relation between ABRIS results and the narrow-band EFR response in its part that displayed the highest difference between the groups, we conducted an ANOVA test with factors of ABRIS result (negative–“refer” or positive–“pass”) and FreqDiag score. We chose a 2 x 2 between-subjects robust aligned rank transform test ANOVA due to violations of normality and non-homogeneity of variances in the untransformed data. We observed the significant ABRIS x FreqDiag interaction *F*_(1,50)_ = 5.57, *p* < 0.05. The marginal means plot is depicted in Figure 4A. The main effect of FreqDiag was absent *F*_(1,50)_ = 1.69, *p* = 0.2, but there was a significant main effect of ABRIS result *F*_(1,46)_ = 7.69, *p* < 0.01. Note that the validity of results is constrained by the strongly unbalanced design with only three observations of MCSe patients with negative ABRIS results (other subgroups MCSe/ABRIS-positive–23 subjects, UWS/ABRIS-negative–11 subjects, UWS/ABRIS-positive–17 subjects). The inspection of the plot indicates that the ABRIS result did not have an influence on UWS results and, in accordance with our suspicions, it probably had an impact on the MCSe group, substantially decreasing ITPC levels in the negative ABRIS subgroup to the level obtained by the UWS group (note, however, the previous remark on the number of subjects, and extensive CI range for the MCSe/ABRIS negative group).

### 3.3 Wide-band chirp condition—Effects of diagnosis

**FIGURE 5:**
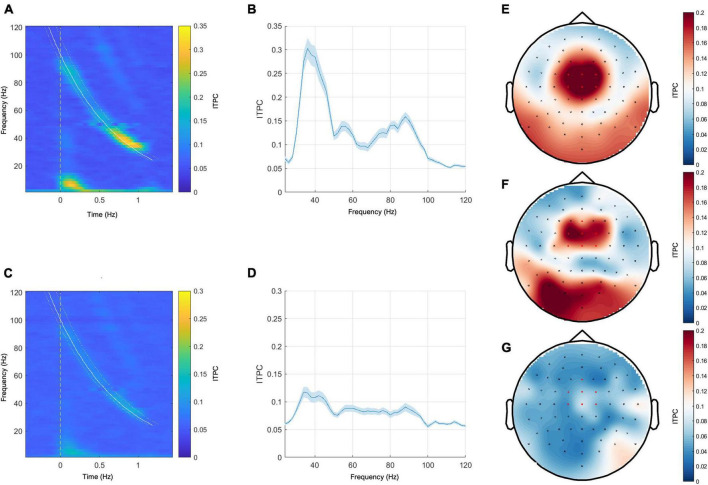
Left panel: grand mean responses to wide-band chirp-modulated stimulation on the time-frequency plane in the healthy control group **(A)** and in the patient group **(C)**. Middle panel: grand mean EFR curves over frequency in the healthy control group **(B)** and the patient group **(D)**. The blue ribbons represent the standard error of the mean. Right panel: topoplots for the wide-band chirp condition ITPC response sampled from the range of 40–50 Hz. **(E)** Grand mean response of the control group, **(F)** representative subject with the MCS+ diagnosis and the positive ABRIS result, **(G)** representative subject with UWS diagnosis and the positive ABRIS result. Red-colored dots indicate channels that were included in calculating EFR responses.

The grand mean responses for the wide-band chirp stimulation condition for the control group and the patients are shown in Figure 5. The highest ITPC response can be observed around 40 Hz (low-gamma band) in both groups. The peak around the high-gamma band can be readily observed in the control group, while it is much smaller in the patient group. The response topographies in the low-gamma (range 40–50 Hz) in Figure 5 (the right panel) indicate that the response was most pronounced in the frontocentral channels and was visible in controls and the representative patient from the aware group and was barely visible in the representative case from the unaware group. The outlier detection procedure did not exclude any patients in the wide-band chirp condition.

**FIGURE 6:**
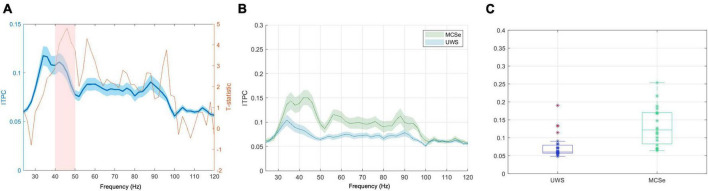
The wide-band chirp condition results indicating differences in low-gamma response between aware and unaware groups (FreqDiag variable). **(A)** Results of the non-parametric cluster-level statistical analysis for the differences between MCSe and UWS groups, blue plot: grand average EFR response, with overlaid T-statistic scores (red plot), the pink box represents the suprathreshold cluster, **(B)** mean EFR curves for both FreqDiag groups; **(C)** box-plots of group FreqDiag results with individual data, extreme values indicated in red. Ribbons on panels **(A,B)** represent the standard error of the mean.

The non-parametric cluster-based permutation procedure with FreqDiag as the independent variable did not reveal any suprathreshold cluster at *p* < 0.001. However, at a more relaxed threshold *p* < 0.005, a significant difference in wide-band EFR response between both patient groups was revealed, corresponding to the cluster at a low gamma range (40–50 Hz, clusterstat = 24.06, *p* < 0.005). The plots depicting statistical scores, the comparison of the EFR responses range, and the individual ITPC scores are depicted in Figure 6. Similarly to the previous condition, the individual results in the UWS group are concentrated between 0.05–0.01, with three cases above 0.1 level. The individual results of the MCSe group display a much greater spread, with higher responses on average and several observations below 0.1 level. See Table 3 for the mean ITPC scores for both patient groups in this condition.

**TABLE 3:** Number of observations across etiology categories, split by the FreqDiag group assignment.

Etiology	UWS	MCSe
Anoxia	14	2
Trauma	7	12
Anoxia and trauma	4	0
Stroke	1	10
Stroke and anoxia	1	0
Other etiology	1	1

UWS, the unaware group; MCSe, the aware group.

### 3.4 Wide-band chirp condition—Effects of auditory pathway integrity

In order to remove the influence of the factor of auditory pathway integrity, we conducted the non-parametric cluster-based analysis constrained to the subjects with positive ABRIS results. Again, there was not any significant difference at *p* < 0.001. Still, a significant difference was observed at the relaxed *p* < 0.005 threshold, corresponding to the suprathreshold cluster spanning the 42–50 Hz range (*N* = 35, clusterstat = 19.08, *p* < 0.005).

**FIGURE 7:**
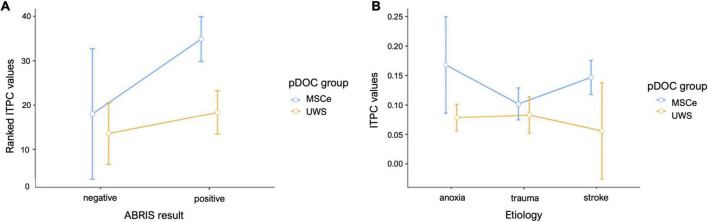
Mean plots for the data sampled from the suprathreshold cluster in the wide-band chirp condition. **(A)** Mean results for the FreqDiag groups split by ABRIS screening test results, **(B)** mean results for the FreqDiag groups split by etiology category. Error bars represent 95% confidence intervals.

To obtain a more detailed view of the possible interaction between the factor of auditory pathway integrity and pDOC diagnosis, we analyzed mean ITPC scores aggregated from the suprathreshold cluster data. Similarly to the narrow-band stimulation, we performed the aligned rank transform test ANOVA (due to violations of the ordinary ANOVA assumptions) using a 2 × 2 design. The interaction of ABRIS results and FreqDiag group was insignificant *F*_(1,42)_ = 1.90, *p* = 0.176, and at the same time, both the main effect of ABRIS result and FreqDiag diagnosis were significant [*F*_(1,42)_ = 6.07, *p* < 0.05 and *F*_(1,42)_ = 5.70, *p* < 0.05, respectively]. The marginal means plot is shown in Figure 7A. Similarly to the results in the narrow-band chirp condition, the current result must be interpreted with caution because of the non-balanced design (MCSe/ABRIS-positive–17 subjects, MCSe/ABRIS-negative–2 subjects, UWS/ABRIS-positive–18 subjects, UWS/ABRIS-negative–9 subjects). Nevertheless, the current results show that negative ABRIS result decreases the response in the MCSe group and has lesser influence in the UWS group, though it is more pronounced than in the previous condition and reduces the interaction effect. The results for the wide-band stimulation show a less systematic relationship between evoked EFR response to wide-band stimulation and the pDOC diagnosis. Nevertheless, just as for the narrow-band chirp condition, the low-gamma response appears as the most sensitive part of the response to the most frequent diagnosis.

### 3.5 Narrow-band chirp condition—Effects of etiology

We also attempted to examine the potential effects of the cause of brain injury on the EFR responses in the studied group of pDOC patients. Table 3 shows the sizes of patient subgroups in the etiology categories we have distinguished. We have compared responses across the etiologies with the highest numbers of included observations, namely, anoxia, trauma, and stroke. We have compared average ITPC values sampled from the suprathreshold cluster obtained for the FreqDiag analysis described previously. The results showed that the most pronounced difference between FreqDiag groups was observed within the stroke group, with other groups having smaller differences between groups (see Figure 4B). Notably, within the etiology category of trauma, the differences between both groups were reduced, seemingly due to the heightened ITPC response in the UWS subgroup.

### 3.6 Wide-band chirp condition—Effects of etiology

For the wide-band chirp condition we had, as for the previous type of stimulation, the unbalanced size of subgroups prevented performing strict statistical tests (see Table 3). As for the previous stimulation type, we focused on the three etiologies with the highest group sizes. We compared responses in the low-gamma cluster identified by the non-parametric cluster test for the FreqDiag independent variable (see Figure 7B). Similarly to the previous simulation, the trauma group showed the smallest difference between pDOC groups, this time this reduction was due to lowered responses in the MSCe group. In other etiology groups, MCSe patients on average displayed higher ITPC responses in the low-gamma band.

Overall, despite the unbalanced group size, the results suggest that the etiology of the brain injury may have considerable influence on the responsitivity to the chirp-based auditory stimulation in the studied pDOC group. Three points can be inferred. The first is a relatively stable and high response in the group of MCSe stroke patients for both types of stimulation. On the other extreme, UWS patients with anoxic etiology showed systematically virtually absent response to stimulation. The third point is related to the results obtained in the trauma group for which the difference between the UWS and MCSe was least pronounced. These issues will be further discussed in the section “4 Discussion” of the paper.

## 4 Discussion

The aim of this study was to assess the sensitivity of EFR response to chirp-modulated stimulation in a group of patients with prolonged Disorders of Consciousness. We have chosen two types of chirp-modulated stimulation—narrow-band stimulation centered around 40 Hz and based on amplitude modulation and wide-band stimulation, covering both low-gamma and high-gamma frequency ranges, based on a series of clicks. The pDOC diagnosis, representing the level of consciousness, was based on the repeated CRS-R evaluation (Seel et al., 2010; Giacino et al., 2018). CRS-R is regarded as a “gold standard” in the assessment of pDOC, and multiple administration has been proven to lower the risk of misdiagnosis (Wannez et al., 2017). As there is no consensus in the literature concerning the integration of multiple CRS-R assessments into a single diagnostic score, we chose a way of parametrizing the diagnosis of pDOC based on the dominant diagnosis across five measurements. This approach emphasizes the potential of the diagnosed patient to manifest behavioral markers of the respective diagnostic entity. We decided not to choose the parameter based on the best diagnosis, as we see it as posing the risk of amplifying a single misdiagnosis over the whole assessment series if it happens to be the best one.

We used a 25–55 Hz frequency range in the first condition to explore the low-gamma response. We chose it as it is known that the maximum response frequency in the low-gamma range is subject to some individual variation (Mockevičius et al., 2023), and thus using many frequencies instead of a single one may create an opportunity to capture the maximal low-gamma response in all studied subjects. We decided to represent the evoked response by the EFR curve because it accurately represents the dynamic changes in the sensitivity of the ITPC response across time and frequency. The grand average response in a group of 20 healthy subjects was represented by the gradual increase in ITPC with a maximal response at around 40 Hz. The topographic plot of the response around its maximum (range 32–50 Hz) shows that the maximal response was observed in the frontocentral channels. Such topographic distribution has been reported in other studies employing chirp-modulated stimulation involving low-gamma (Pipinis et al., 2018), and the studies involving constant 40 Hz stimulation (Ross, 2013). This supports the conjecture that the observed response has been generated in the auditory cortex and probably other sources that are involved in 40 Hz ASSR generation (Farahani et al., 2017, 2019, 2021). A similar profile, as well as the maximal frequency of response, was observed in the pDOC patient group (see Figure 2B for the healthy control group and Figure 2D for the patient group), albeit the consistency of the response was notably lower than in the control group.

In the case of the wide-band stimulation, we were interested in the effects of the sensitivity to the stimulation frequencies beyond low gamma, focusing primarily on high gamma as the other potential source of meaningful differences among pDOC patient groups. We decided not to include lower frequencies, firstly, because they coincide with high-amplitude physiological oscillations that may substantially lower signal-to-noise ratio, and secondly, because the appropriate estimation of lower frequencies requires longer inter-stimulus intervals and would make our protocol considerably longer and thus harder to implement, especially in the challenging conditions of pDOC patient measurement. The control group results showed the dominant EFR response within the low-gamma range (see Figures 5A, B). In the case of pDOC patients, the low-gamma response was greatly diminished (see Figures 5C, D). The topography of the low-gamma was centered around the frontocentral channels, just as in the case of narrow-band stimulation (see Figure 5, the right panel).

The analysis of the narrow-band chirp condition results revealed the low-gamma cluster that was also visible in the same location in the reduced group with positive ABRIS results. This result corroborates the conclusion that the low-gamma response depends on the response originating in the upper parts of the auditory system whose function is somehow connected to the pDOC status. The ANOVA analysis testing for interaction between the FreqDiag group factor and ABRIS result, on the other hand, suggests that the negative ABRIS results (indicating disruption of the brainstem parts of the auditory pathway) may substantially reduce the EFR low-gamma response in the MCSe group. Observations not aligned with the central tendency suggest that factors other than brainstem integrity may influence response variability in the pDOC group. One of them may be the changed topography of the response caused by changed dipole orientation resulting from structural damage to the neural tissue. In this case, the highest response will be observed beyond the seven channels we have selected. Indeed, analysis of the influence of etiology on the effect of the narrow-band stimulation suggested that trauma etiology may change the pattern of results within the group, where the diagnosis of pDOC did not differentiate between EFR response. The structural damage and the dipole orientation reflected in the changed topography may at least partly explain that result.

The wide-band chirp condition analysis of EFR response revealed a low-gamma cluster both in the analysis including all suitable patients and in the analysis limited to the group of the ABRIS positive patients, though using a more lenient significance threshold. The influence of etiology on the results obtained in this cluster was similar to the narrow-band chirp condition, with the traumatic group not showing a clear relationship between the FreqDiag score and the ITPC result. As in the previous condition, in the most numerous Etiology subgroups (despite lack of balance), in the anoxia group, low FreqDiag scores coincided with low ITPC, and reversely, in the stroke group, high FreqDiag scores coincided with high ITPC scores.

In conclusion, the results of both stimulation conditions suggest that the low-gamma response to periodic auditory stimulation measured from the frontocentral channels exhibits sensitivity to the ability of pDOC patients to manifest signs of awareness as measured with multiple CRS-R administration. On the one hand, this sensitivity manifested as a very diminished response in all patients with unfavorable FreqDiag scores. On the other hand, the patients that scored higher had elevated EFR responses, yet they displayed some variability, which at least in part can be accounted for by the etiology of their brain injury with less meaningful responses from the subjects with traumatic brain injury.

The response in other frequency bands showed no significant relationship with the awareness diagnosis. The preliminary data from other experiments with broad-band stimulations (Górska and Binder, unpublished data) suggest higher ITPC at the high-gamma band in conditions of low arousal (NREM sleep, general anesthesia), which is probably caused by disinhibition of that response, yet they may be related to other brain mechanisms. The lack of a systematic relationship in other frequencies suggests that the low-gamma response cannot be attributed to generalized changes in auditory cortex responsitivity to auditory stimulation across all stimulation frequencies but suggests a more selective type of response—pointing to the specific mechanism that may become severely disrupted in the unaware pDOC patients.

As to the possible account for the observed effects, there is evidence that the disruption of low-gamma response can be treated as the marker of disrupted excitation-inhibition balance (E/I balance) across the cortical mantle (Tada et al., 2020; Ahmad et al., 2022). According to Tada et al. (2020), the entrainment hypothesis of 40 Hz stimulation is based on the endogenous oscillatory activity in the gamma range based on the interaction between GABAergic interneurons and pyramidal excitatory neurons or based mainly on the inhibitory PV+ networks activity. Low-gamma responses to auditory stimulation are widely seen as the selective marker of the ability to maintain this E/I balance, which is crucial for the efficient functioning of the cortex. Although this account has been mainly used to explain differences in 40 Hz ASSR responses in neuropsychiatric disorders, predominantly schizophrenia, which are relatively small in comparison to the effects observed in the current study, it may nevertheless point to the meaningful connection between the E/I capacity and the networks underlying awareness. In this light, the proposed protocols can be utilized as the perturbational markers of the E/I balance.

Another explanation, which does not exclude the previous one, is based on the general disruption of the arousal networks that are supplied by the centers in the dorsal brainstem and central thalamus (Schiff, 2010). The low-gamma responses are known to depend on the cholinergic system (Zhang et al., 2016) and glutaminergic NMDA receptors (Sivarao, 2015; Sivarao et al., 2016). Disruptions of those systems may also be present in pDOC and play a significant role in influencing the strength and consistency of the low-gamma response in the studied group of pDOC patients.

Unfortunately, we could not obtain balanced sizes in all etiology categories. Such distribution stems from the fact that different etiologies tend to co-occur with specific pDOC diagnoses, which is caused by the fact that depending on the etiology, the brain injury associated with it disrupts the structure and the function of the central nervous system in an unequal way, and in case anoxic etiology usually the extent of the damage is more extensive than in the case of stroke or traumatic injury. This effect is strengthened in time, following several months after the incident, because different etiologies also differ with respect to the rate of recovery. From the statistical point of view, this size imbalance makes it impossible to perform a strict statistical analysis of the effects of etiology. Thus, the conclusions are tentative.

The main limitation of the current study is the size of the studied patient sample, which needs to be bigger to perform a statistically sound comparison of the groups depending on their etiology. The imbalance of the group sizes is also because our subjects were patients with prolonged DOC, which means that the different recovery rates depending on the cause of brain injury were reflected in the availability of subjects across different etiology groups, e.g., patients with anoxic etiology prevailing in the UWS group. Nevertheless, the observed tendencies represent a reliable indication of the effects of etiology and thus set up a good starting point for a follow-up study exploring in a more systematic way low-gamma responses across various types of brain injury.

## 5 Conclusion

In this study, we examined the responsiveness of the auditory system using the Envelope Following Response in a group of patients with prolonged Disorders of Consciousness with differing diagnoses and etiologies of brain injury. We applied two types of periodic chirp-modulated auditory stimulation: amplitude-modulated narrow-band stimulation (25–55 Hz) and click-based wideband stimulation (30–100 Hz). We used the temporal-frequency changes in the intertrial phase clustering coefficient following frequency changes as a response parameter, which was presented as an EFR curve.

For both types of stimulation, we observed variations in the strength of the response in the low-gamma range, which were related to the prevailing diagnosis of pDOC. We observed diminished responses in patients diagnosed with unresponsive wakefulness syndrome, while patients with more favorable diagnoses showed more pronounced responses. The integrity of the auditory pathway and the etiology of brain injury exerted a modifying influence on the observed response strength, with negative ABR results and traumatic etiology associated with decreased responses in the low-gamma range in the aware group. Narrow-band stimulation yielded a more systematic relationship between low-gamma response and pDOC diagnosis.

The results of the study suggest that measuring EFR responses in the low-gamma range can be used as a supportive tool for diagnosing pDOC. Detection of low or absent responses may suggest an unaware state of the brain, while higher responses may indicate an aware state. However, due to the observed variability of results, caution should be exercised when interpreting negative effects (risk of false negatives, low specificity), while positive effects may have diagnostic value.

Auditory responses observed in our study may provide the basis for constructing a relevant set of features for the machine learning models that can be used for improved diagnosis and prediction of patients’ outcomes (Mofatteh, 2021; Liuzzi et al., 2022). Our approach could prove particularly advantageous in large-scale studies, where it is highly suitable for integration.

## Data availability statement

The datasets presented in this study can be found in online repositories. The names of the repository/repositories and accession number(s) can be found below: https://osf.io/xq485/?view_only=e67da8690c36483e84e8bccdb5737e1d.

## Ethics statement

The studies involving humans were approved by the Research Ethics Review Board, Institute of Psychology, Jagiellonian University. The studies were conducted in accordance with the local legislation and institutional requirements. Written informed consent for participation in this study was provided by the participants’ legal guardians/next of kin.

## Author contributions

MB: conceptualization, methodology, software, formal analysis, investigation, data curation, writing–original draft, writing–review and editing, visualization, supervision, project administration, and funding acquisition. JP: investigation, data curation, writing–original draft, and writing–review and editing. IG-B: conceptualization, methodology, formal analysis, and writing–review and editing. SF: investigation, data curation, writing–original draft, and writing–review and editing. BC: methodology, resources, and writing–original draft. UG-K: conceptualization, methodology, and writing–review and editing. All authors contributed to the article and approved the submitted version.
