# Supplementary material for: Diagnosing awareness in disorders of consciousness with gamma-band auditory responses
Source: Front Hum Neurosci. 2024 Jan 5;17:1243051. doi: 10.3389/fnhum.2023.1243051 (PMC10796678; doi:10.3389/fnhum.2023.1243051)
Supplement: Supplementary file 1 [file Table_1.pdf]

Table A1. Demographic details of the healthy control group

ID	Group	Sex	Age	Handedness	OAE screening	ABR screening
C001	HC	M	44	R	1	1
C002	HC	F	35	no data	1	1
C003	HC	F	22	L	1	1
C004	HC	F	20	L	1	1
C005	HC	F	22	R	1	1
C006	HC	F	33	R	1	1
C007	HC	F	55	R	1	1
C008	HC	M	22	R	1	1
C009	HC	M	24	R	1	1
C010	HC	M	41	R	1	1
C011	HC	F	30	R	1	1
C012	HC	F	20	R	1	1
C013	HC	M	33	R	1	1
C014	HC	F	29	R	1	1
C015	HC	M	33	R	1	1
C016	HC	M	27	R	1	1
C017	HC	M	26	R	1	1
C018	HC	M	29	R	1	1
C019	HC	M	23	R	1	1
C020	HC	M	21	R	1	1

HC – healthy control, *F* – female, *M* – male, *R* – right, *L* – left, *OAE* – otoacoustic emissions, *ABR* – auditory brainstem response

Note. All participants from the HC group were included for further analysis of EEG data both in narrow-band chirp condition and wide-band chirp condition.
