# Supplementary material for: Diagnosing awareness in disorders of consciousness with gamma-band auditory responses
Source: Front Hum Neurosci. 2024 Jan 5;17:1243051. doi: 10.3389/fnhum.2023.1243051 (PMC10796678; doi:10.3389/fnhum.2023.1243051)
Supplement: Supplementary file 2 [file Table_2.pdf]

Patient ID	Age	Gender	Time since injury in months	Etiology	1st CRS-R assessment		2nd CRS-R assessment		3rd CRS-R assessment		4th CRS-R assessment		5th CRS-R assessment		Most frequent diagnosis
					Subscores	Final diagnosis	Subscores	Final diagnosis	Subscores	Final diagnosis	Subscores	Final diagnosis	Subscores	Final diagnosis	
01 (58)	34	M	67	trauma, anoxia	A1 V0 M1 O/V1 C0 Ar2	UWS	A1 V0 M2 O/V1 C0 Ar2	UWS	A1 V0 M2 O/V1 C0 Ar2	UWS	A1 V1 M1 O/V1 C0 Ar2	UWS	A1 V0 M2 O/V1 C0 Ar2	UWS	UWS
02 (59)	29	F	66	trauma	A1 V0 M2 O/V1 C0 Ar1	UWS	A1 V0 M2 O/V1 C0 Ar1	UWS	A1 V1 M2 O/V1 C0 Ar1	UWS	A3 V1 M5 O/V1 C1 Ar2	MCS+	A1 V1 M2 O/V1 C1 Ar1	MCS+	UWS
03 (99)	37	M	64	anoxia	A1 V1 M2 O/V1 C0 Ar2	UWS	A1 V1 M2 O/V1 C0 Ar1	UWS	A1 V1 M2 O/V1 C0 Ar2	UWS	A1 V0 M1 O/V1 C0 Ar1	UWS	A1 V1 M2 O/V1 C0 Ar1	UWS	UWS
04 (111)	41	M	43	anoxia	A1 V1 M2 O/V1 C0 Ar2	UWS	A1 V0 M1 O/V0 C0 Ar1	UWS	A1 V0 M1 O/V1 C0 Ar1	UWS	A1 V1 M2 O/V1 C0 Ar1	UWS	A1 V0 M2 O/V2 C0 Ar1	UWS	UWS
05 (119)	27	M	11	trauma	A2 V1 M1 O/V1 C0 Ar2	UWS	A1 V1 M2 O/V2 C0 Ar2	UWS	A1 V1 M1 O/V0 C0 Ar2	UWS	A1 V2 M2 O/V1 C0 Ar1	MCS-	A1 V1 M2 O/V1 C0 Ar1	UWS	UWS
06 (121)	21	M	7	trauma	A1 V0 M1 O/V1 C0 Ar1	UWS	A1 V0 M0 O/V0 C0 Ar0	UWS	A1 V0 M0 O/V1 C0 Ar0	UWS	A4 V1 M1 O/V1 C0 Ar0	MCS+	A1 V1 M2 O/V2 C0 Ar0	UWS	UWS
07 (122)	28	M	4	trauma	A1 V1 M0 O/V1 C0 Ar2	UWS	A1 V0 M0 O/V1 C0 Ar2	UWS	A1 V0 M0 O/V1 C0 Ar2	UWS	A3 V0 M1 O/V1 C0 Ar1	MCS+	A1 V1 M1 O/V1 C0 Ar1	UWS	UWS
08 (127)	48	M	7	anoxia	A1 V0 M1 O/V1 C0 Ar0	UWS	A1 V0 M1 O/V1 C0 Ar2	UWS	A1 V0 M1 O/V0 C0 Ar1	UWS	A1 V1 M1 O/V0 C0 Ar1	UWS	A1 V0 M1 O/V0 C0 Ar2	UWS	UWS
09 (129)	59	M	2	stroke	A1 V1 M2 O/V1 C0 Ar1	UWS	A1 V0 M1 O/V1 C0 Ar0	UWS	A1 V0 M2 O/V1 C0 Ar0	UWS	A1 V1 M2 O/V1 C0 Ar1	UWS	A0 V0 M2 O/V1 C0 Ar1	UWS	UWS
10 (132)	48	M	9	anoxia	A1 V0 M1 O/V1 C0 Ar0	UWS	A1 V0 M1 O/V1 C0 Ar0	UWS	A1 V1 M2 O/V0 C0 Ar1	UWS	A1 V1 M0 O/V0 C0 Ar2	UWS	-	-	UWS
11 (143)	56	M	11	anoxia	A1 V1 M0 O/V1 C0 Ar1	UWS	A1 V0 M1 O/V1 C0 Ar1	UWS	A1 V0 M0 O/V1 C0 Ar1	UWS	A1 V1 M0 O/V1 C0 Ar1	UWS	A1 V1 M0 O/V1 C0 Ar1	UWS	UWS
12 (144)	36	F	33	other	A1 V0 M1 O/V1 C0 Ar1	UWS	A1 V1 M2 O/V2 C0 Ar2	UWS	A3 V1 M2 O/V1 C1 Ar1	MCS+	A1 V1 M2 O/V1 C0 Ar2	UWS	A1 V0 M2 O/V1 C0 Ar2	UWS	UWS
13 (145)	40	F	23	anoxia	A1 V1 M1 O/V1 C0 Ar1	UWS	A1 V0 M1 O/V1 C0 Ar2	UWS	A1 V0 M2 O/V1 C0 Ar2	UWS	A1 V0 M2 O/V1 C0 Ar1	UWS	A1 V1 M2 O/V1 C0 Ar1	UWS	UWS
14 (147)	65	F	45	anoxia	A1 V1 M3 O/V1 C0 Ar1	UWS	A1 V1 M0 O/V1 C0 Ar1	UWS	A2 V1 M0 O/V1 C0 Ar1	UWS	A2 V1 M2 O/V1 C0 Ar1	UWS	A1 V1 M1 O/V1 C0 Ar2	UWS	UWS
15 (148)	42	M	20	anoxia	A1 V0 M2 O/V1 C0 Ar1	UWS	A1 V0 M1 O/V1 C0 Ar0	UWS	A1 V1 M0 O/V1 C0 Ar1	UWS	A1 V0 M1 O/V2 C1 Ar0	UWS	A1 V1 M1 O/V1 C0 Ar1	UWS	UWS
16 (149)	35	M	35	anoxia	A1 V0 M0 O/V1 C0 Ar2	UWS	A1 V0 M0 O/V1 C0 Ar2	UWS	A1 V1 M2 O/V1 C0 Ar2	UWS	A1 V0 M2 O/V1 C0 Ar2	UWS	A1 V0 M2 O/V1 C0 Ar2	UWS	UWS
17 (150)	30	M	20	trauma, anoxia	A3 V1 M0 O/V1 C0 Ar1	MCS+	A1 V1 M2 O/V2 C0 Ar2	UWS	A1 V1 M2 O/V1 C0 Ar2	UWS	A3 V0 M1 O/V1 C0 Ar2	MCS+	A1 V1 M0 O/V1 C0 Ar1	UWS	UWS
18 (151)	54	M	31	anoxia	A0 V0 M0 O/V1 C0 Ar1	UWS	A1 V0 M1 O/V1 C0 Ar2	UWS	A1 V0 M1 O/V1 C0 Ar1	UWS	A1 V0 M0 O/V1 C0 Ar1	UWS	A1 V0 M2 O/V1 C0 Ar1	UWS	UWS
19 (154)	48	M	25	anoxia	A1 V0 M2 O/V2 C0 Ar2	UWS	A0 V1 M1 O/V1 C0 Ar2	UWS	A1 V0 M2 O/V1 C0 Ar1	UWS	A1 V0 M2 O/V1 C0 Ar1	UWS	A1 V0 M2 O/V1 C0 Ar1	UWS	UWS
20 (159)	19	M	4	anoxia	A1 V0 M0 O/V0 C0 Ar2	UWS	A0 V0 M0 O/V1 C0 Ar2	UWS	A1 V0 M1 O/V0 C0 Ar2	UWS	A1 V1 M1 O/V1 C0 Ar2	UWS	A1 V0 M0 O/V1 C0 Ar2	UWS	UWS
21 (165)	44	F	4	anoxia	A1 V0 M0 O/V1 C0 Ar1	UWS	A1 V0 M0 O/V1 C0 Ar2	UWS	A1 V1 M1 O/V1 C0 Ar1	UWS	A1 V1 M2 O/V1 C0 Ar2	UWS	A1 V1 M1 O/V1 C0 Ar2	UWS	UWS
22 (166)	62	F	3	stroke, anoxia	A1 V1 M0 O/V1 C0 Ar1	UWS	A1 V0 M1 O/V1 C0 Ar0	UWS	A1 V1 M2 O/V1 C0 Ar1	UWS	A1 V1 M2 O/V1 C0 Ar1	UWS	A1 V0 M2 O/V0 C0 Ar2	UWS	UWS
23 (168)	38	F	5	anoxia	A1 V0 M1 O/V0 C0 Ar1	UWS	A1 V0 M1 O/V1 C0 Ar1	UWS	A1 V0 M0 O/V0 C0 Ar1	UWS	A1 V0 M1 O/V1 C0 Ar1	UWS	A1 V0 M1 O/V0 C0 Ar1	UWS	UWS
24 (170)	43	F	2	trauma, anoxia	A2 V1 M2 O/V0 C0 Ar2	UWS	A1 V0 M1 O/V1 C0 Ar2	UWS	A2 V0 M1 O/V1 C0 Ar1	UWS	A1 V0 M1 O/V0 C0 Ar2	UWS	A1 V0 M1 O/V0 C0 Ar1	UWS	UWS
25 (171)	37	M	6	trauma	A1 V0 M1 O/V1 C0 Ar1	UWS	A1 V1 M2 O/V1 C0 Ar1	UWS	A1 V0 M1 O/V1 C0 Ar1	UWS	A1 V0 M2 O/V1 C0 Ar1	UWS	A1 V0 M1 O/V1 C0 Ar1	UWS	UWS
26 (172)	18	F	24	trauma, anoxia	A1 V0 M0 O/V1 C0 Ar2	UWS	A1 V0 M2 O/V1 C0 Ar2	UWS	A1 V0 M2 O/V1 C0 Ar2	UWS	A1 V0 M1 O/V1 C0 Ar2	UWS	A0 V1 M1 O/V1 C0 Ar1	UWS	UWS
27 (173)	59	M	10	trauma	A1 V1 M1 O/V1 C0 Ar2	UWS	A1 V1 M1 O/V1 C0 Ar1	UWS	A1 V1 M1 O/V1 C0 Ar0	UWS	A1 V1 M1 O/V1 C0 Ar2	UWS	A0 V1 M1 O/V1 C0 Ar1	UWS	UWS
28 (174)	65	M	8	anoxia	A1 V0 M1 O/V1 C0 Ar1	UWS	A0 V0 M1 O/V1 C0 Ar1	UWS	A0 V0 M1 O/V1 C0 Ar1	UWS	A1 V0 M2 O/V1 C0 Ar1	UWS	A1 V1 M1 O/V1 C0 Ar1	UWS	UWS
29 (175)	74	F	25	trauma	A0 V0 M2 O/V1 C0 Ar2	UWS	A0 V0 M1 O/V1 C0 Ar1	UWS	A1 V3 M1 O/V1 C0 Ar2	MCS-	A1 V1 M1 O/V1 C0 Ar1	UWS	A0 V3 M2 O/V1 C0 Ar2	MCS-	UWS
30 (177)	22	M	26	trauma, anoxia	A1 V0 M1 O/V1 C0 Ar1	UWS	A1 V0 M1 O/V0 C0 Ar1	UWS	A1 V0 M2 O/V1 C0 Ar1	UWS	A1 V0 M1 O/V1 C0 Ar1	UWS	A1 V0 M1 O/V1 C0 Ar2	UWS	UWS
31 (184)	34	F	10	anoxia	A0 V0 M2 O/V0 C0 Ar1	UWS	A1 V0 M2 O/V0 C0 Ar1	UWS	A0 V0 M2 O/V0 C0 Ar2	UWS	A1 V1 M1 O/V1 C0 Ar1	UWS	A1 V1 M1 O/V0 C0 Ar2	UWS	UWS
32 (195)	45	M	11	anoxia	A1 V0 M0 O/V1 C0 Ar2	UWS	A1 V1 M1 O/V1 C0 Ar1	UWS	A1 V0 M1 O/V1 C0 Ar1	UWS	A1 V0 M1 O/V1 C0 Ar2	UWS	A2 V1 M1 O/V1 C0 Ar1	UWS	UWS
33 (117)	20	M	23	trauma	A3 V3 M1 O/V1 C0 Ar2	MCS-	A1 V3 M0 O/V2 C0 Ar2	MCS-	A1 V1 M1 O/V1 C0 Ar2	UWS	A1 V1 M1 O/V2 C0 Ar2	UWS	A1 V3 M0 O/V2 C0 Ar2	MCS-	MCS-
34 (120)	30	M	9	trauma	A1 V3 M0 O/V1 C0 Ar2	MCS-	A1 V3 M1 O/V1 C0 Ar2	MCS-	A2 V3 M0 O/V1 C0 Ar3	MCS-	A2 V3 M0 O/V2 C0 Ar2	MCS-	A2 V3 M0 O/V1 C0 Ar1	MCS-	MCS-
35 (134)	30	M	7	stroke	A1 V3 M5 O/V2 C0 Ar1	MCS-	A1 V3 M1 O/V2 C0 Ar1	MCS-	A1 V3 M1 O/V1 C0 Ar1	MCS-	A1 V3 M0 O/V2 C0 Ar1	MCS-	A1 V3 M0 O/V1 C0 Ar1	MCS-	MCS-
36 (138)	34	M	17	trauma	A3 V5 M2 O/V1 C0 Ar2	MCS+	A2 V3 M1 O/V2 C0 Ar1	MCS-	A1 V1 M2 O/V0 C0 Ar1	UWS	A2 V3 M2 O/V1 C0 Ar2	MCS-	A1 V3 M2 O/V1 C0 Ar2	MCS-	MCS-
37 (140)	39	M	2	stroke	A2 V4 M5 O/V1 C0 Ar2	MCS-	A3 V5 M5 O/V2 C0 Ar2	MCS+	A1 V3 M5 O/V2 C0 Ar2	MCS-	A1 V4 M5 O/V2 C0 Ar3	MCS-	A1 V4 M5 O/V2 C0 Ar3	MCS-	MCS-
38 (155)	48	M	3	stroke	A2 V4 M5 O/V2 C0 Ar2	MCS-	A3 V4 M5 O/V3 C0 Ar2	MCS+	A2 V3 M5 O/V1 C0 Ar2	MCS-	A2 V3 M5 O/V1 C0 Ar2	MCS-	A2 V3 M5 O/V2 C0 Ar2	MCS-	MCS-
39 (158)	40	M	11	other	A0 V0 M0 O/V0 C0 Ar2	UWS	A0 V2 M2 O/V1 C0 Ar1	MCS-	A0 V3 M1 O/V2 C0 Ar1	MCS-	A0 V3 M5 O/V1 C0 Ar1	MCS-	A3 V3 M4 O/V0 C1 Ar2	MCS+	MCS-
40 (160)	51	M	10	trauma	A0 V0 M2 O/V1 C0 Ar0	UWS	A2 V3 M2 O/V1 C0 Ar1	MCS-	A0 V3 M2 O/V1 C0 Ar1	MCS-	A2 V3 M2 O/V2 C0 Ar2	MCS-	A2 V3 M0 O/V2 C1 Ar2	MCS+	MCS-
41 (163)	28	M	14	trauma	A1 V2 M1 O/V1 C0 Ar2	MCS-	A1 V3 M2 O/V1 C0 Ar2	MCS-	A1 V3 M2 O/V1 C0 Ar2	MCS-	A3 V3 M2 O/V1 C0 Ar2	MCS+	A1 V4 M2 O/V2 C1 Ar2	MCS+	MCS-
42 (167)	46	M	5	trauma	A2 V1 M1 O/V0 C0 Ar2	UWS	A1 V3 M1 O/V1 C0 Ar1	MCS-	A1 V1 M1 O/V1 C0 Ar1	UWS	A1 V3 M1 O/V1 C0 Ar1	MCS-	A1 V3 M2 O/V0 C0 Ar1	MCS-	MCS-
43 (169)	53	F	7	stroke	A0 V3 M5 O/V1 C0 Ar2	MCS-	A1 V4 M5 O/V1 C0 Ar2	MCS-	A1 V4 M5 O/V1 C0 Ar2	MCS-	A1 V4 M6 O/V1 C0 Ar2	EMCS	A1 V4 M0 O/V1 C0 Ar2	MCS-	MCS-
44 (180)	28	M	11	trauma	A1 V3 M1 O/V1 C0 Ar1	MCS-	A1 V3 M1 O/V2 C0 Ar1	MCS-	A0 V0 M2 O/V2 C0 Ar1	UWS	A1 V3 M5 O/V2 C0 Ar2	MCS-	A3 V5 M2 O/V1 C1 Ar1	MCS+	MCS-
45 (181)	44	M	10	anoxia	A0 V3 M1 O/V0 C0 Ar1	MCS-	A1 V1 M1 O/V1 C0 Ar2	UWS	A1 V3 M3 O/V1 C0 Ar2	MCS-	A1 V3 M2 O/V1 C0 Ar2	MCS-	A1 V1 M2 O/V2 C0 Ar1	UWS	MCS-
46 (189)	57	F	6	trauma	A2 V3 M2 O/V1 C0 Ar2	MCS-	A1 V3 M1 O/V1 C0 Ar2	MCS-	A1 V3 M1 O/V1 C0 Ar2	MCS-	A1 V3 M1 O/V1 C0 Ar2	MCS-	A1 V3 M1 O/V1 C0 Ar2	MCS-	MCS-
47 (191)	29	M	8	trauma	A1 V3 M5 O/V0 C0 Ar2	MCS-	A1 V4 M1 O/V1 C0 Ar1	MCS-	A3 V4 M5 O/V0 C0 Ar1	MCS+	A1 V3 M5 O/V0 C0 Ar1	MCS-	A1 V3 M1 O/V0 C0 Ar2	MCS-	MCS-
48 (192)	45	F	2	stroke	A2 V4 M5 O/V2 C0 Ar2	MCS-	A1 V4 M5 O/V2 C0 Ar2	MCS-	A1 V3 M5 O/V2 C0 Ar2	MCS-	A1 V3 M5 O/V1 C0 Ar2	MCS-	A1 V3 M5 O/V2 C0 Ar2	MCS-	MCS-
49 (197)	67	F	3	trauma, anoxia	A2 V3 M2 O/V1 C0 Ar2	MCS-	A1 V1 M2 O/V1 C0 Ar2	UWS	A1 V3 M1 O/V1 C0 Ar2	MCS-	A1 V4 M5 O/V1 C0 Ar2	MCS-	A3 V3 M5 O/V1 C0 Ar2	MCS-	MCS-
50 (124)	35	M	13	trauma	A0 V3 M0 O/V0 C0 Ar1	MCS-	A1 V3 M0 O/V0 C0 Ar2	MCS-	A2 V3 M5 O/V2 C0 Ar3	MCS-	A4 V4 M0 O/V1 C0 Ar2	MCS+	A2 V3 M0 O/V0 C1 Ar1	MCS+	MCS-
51 (123)	42	M	8	anoxia	A2 V3 M1 O/V2 C0 Ar3	MCS-	A3 V3 M1 O/V2 C0 Ar3	MCS+	A2 V3 M1 O/V1 C0 Ar2	MCS-	A4 V5 M2 O/V2 C0 Ar3	MCS+	A3 V5 M2 O/V1 C1 Ar2	MCS+	MCS+
52 (125)	45	M	20	stroke	A3 V4 M5 O/V0 C1 Ar2	MCS+	A3 V4 M5 O/V3 C0 Ar2	MCS+	A3 V4 M5 O/V0 C1 Ar2	MCS+	A2 V4 M3 O/V5 C0 Ar2	MCS+	A2 V4 M6 O/V1 C1 Ar2	EMCS	MCS+
53 (142)	42	M	7	trauma	A3 V4 M5 O/V3 C1 Ar1	MCS+	A1 V3 M2 O/V2 C1Ar1	MCS+	A2 V1 M5 O/V1 C1 Ar1	MCS+	A3 V5 M5 O/V2 C2 Ar1	MCS+	A3 V3 M5 O/V3 C1 Ar1	MCS+	MCS+
54 (153)	36	M	11	anoxia	A1 V0 M1 O/V1 C0 Ar2	UWS	A3 V0 M1 O/V2 C1 Ar2	MCS+	A1 V1 M0 O/V2 C0 Ar2	UWS	A3 V2 M2 O/V2 C1 Ar2	MCS+	A1 V1 M0 O/V1 C1 Ar2	MCS+	MCS+
55 (186)	47	M	7	stroke	A4 V2 M6 O/V3 C1 Ar2	EMCS	A0 V0 M5 O/V1 C0 Ar1	MCS-	A0 V0 M2 O/V1 C0 Ar1	UWS	A4 V0 M5 O/V2 C0 Ar1	MCS+	A3 V0 M5 O/V1 C1 Ar1	MCS+	MCS+
56 (190)	44	F	5	stroke	A3 V5 M2 O/V1 C1 Ar3	MCS+	A3 V4 M5 O/V1 C0 Ar2	MCS+	A4 V5 M1 O/V1 C1 Ar2	MCS+	A3 V5 M2 O/V1 C1 Ar2	MCS+	A3 V5 M2 O/V1 C2 Ar1	EMCS	MCS+
57 (207)	60	F	5	trauma	A3 V1 M2 O/V2 C1 Ar1	MCS+	A3 V3 M2 O/V1 C0 Ar1	MCS+	A3 V3 M0 O/V2 C1 Ar1	MCS+	A4 V5 M5 O/V2 C1 Ar2	MCS+	A3 V3 M0 O/V2 C2 Ar1	EMCS	MCS+
58 (139)	30	M	12	trauma	A3 V5 M5 O/V3 C1 Ar2	MCS+	A3 V5 M2 O/V2 C1 Ar2	MCS+	A3 V5 M5 O/V3 C1 Ar2	MCS+	A3 V5 M5 O/V2 C1 Ar2	MCS+	A1 V4 M5 O/V3 C1 Ar2	MCS+	MCS+
59 (116)	25	F	34	stroke	A4 V5 M6 O/V3 C1 Ar3	EMCS	A4 V5 M6 O/V2 C1 Ar3	EMCS	A3 V4 M4 O/V2 C1 Ar2	MCS+	A4 V5 M6 O/V3 C1 Ar1	EMCS	A4 V5 M6 O/V3 C1 Ar3	EMCS	EMCS
60 (128)	35	F	7	stroke	A3 V5 M6 O/V3 C1 Ar2	EMCS	A4 V5 M6 O/V3 C2 Ar2	EMCS	A4 V5 M5 O/V3 C2 Ar3	EMCS	A4 V5 M4 O/V3 C1 Ar3	MCS+	A4 V5 M0 O/V3 C1 Ar1	MCS+	EMCS
61 (130)	25	M	11	anoxia	A4 V0 M6 O/V3 C2 Ar3	EMCS	A4 V5 M6 O/V3 C2 Ar3	EMCS	A4 V5 M6 O/V3 C2 Ar3	EMCS	A4 V3 M6 O/V3 C2 Ar2	EMCS	A4 V0 M6 O/V3 C2 Ar2	EMCS	EMCS
62 (133)	36	M	2	stroke	A4 V5 M6 O/V2 C2 Ar3	EMCS	A4 V5 M6 O/V2 C2 Ar3	EMCS	A4 V5 M6 O/V2 C2 Ar3	EMCS	A4 V5 M6 O/V2 C2 Ar3	EMCS	A4 V5 M6 O/V2 C2 Ar3	EMCS	EMCS

Table A2. Demographic and clinical data of pDOC patients

F – female, *M* – male, *A* – auditory, *V* – visual, *M* – motor, *O/V* – oromotor/verbal, *C* – communication, *Ar* – arousal, *UWS* – unresponsive wakefulness syndrome, *MCS-* – minimally conscious state minus, *MCS+* – minimally conscious state plus, *EMCS* – emergence from the minimally conscious state
