# Supplementary material for: Diagnosing awareness in disorders of consciousness with gamma-band auditory responses
Source: Front Hum Neurosci. 2024 Jan 5;17:1243051. doi: 10.3389/fnhum.2023.1243051 (PMC10796678; doi:10.3389/fnhum.2023.1243051)
Supplement: Supplementary file 3 [file Table_3.pdf]

Table A3. Measurements of the acoustic output from Head and Torso simulator (HATS) after the calibration procedure for in-ear headphones tests

Narrow-band chirp stimuli			Wide-band chirp stimuli		
**Measurement no.**	**Left ear, dBA**	**Right ear, dBA**	**Measurement no.**	**Left ear, dBA**	**Right ear, dBA**
1	61.4	60.1	1	58.7	60.0
2	61.8	60.9	2	60.2	60.8
3	60.7	61.3	3	59.1	61.2
4	60.1	61.9	4	59.4	61.2
5	60.5	61.0	5	61.0	59.6
Average	**60.9**	**61.0**	Average	**59.7**	**60.6**
Std. dev.	**0.7**	**0.7**	Std. dev.	**0.9**	**0.7**
